# Syringic Acid Ameliorates Cardiac, Hepatic, Renal and Neuronal Damage Induced by Chronic Hyperglycaemia in Wistar Rats: A Behavioural, Biochemical and Histological Analysis

**DOI:** 10.3390/molecules27196722

**Published:** 2022-10-09

**Authors:** Anwarbaig C. Mirza, Shital S. Panchal, Ahmed A. Allam, Sarah I. Othman, Milan Satia, Sanjay N. Mandhane

**Affiliations:** 1 Department of Pharmacology, Institute of Pharmacy, Nirma University, Sarkhej-Ghandinagar Highway, Ahmedabad 382481, Gujarat, India; 2 Department of Pharmacology, School of Pharmacy, AI’s Kalsekar Technical Campus, Navi Mumbai 410206, Maharashtra, India; 3 Department of Zoology, Faculty of Science, Beni-Suef University, Beni-Suef 62511, Egypt; 4 Department of Biology, College of Science, Princess Nourah Bint Abdulrahman University, Riyadh 11671, Saudi Arabia; 5 Ethicare Clinical Trial Services, Ahmedabad 380015, Gujarat, India; 6 Biological Research Pharmacology Department, Sun Pharma Advanced Research Company Ltd., Vadodara 390010, Gujarat, India

**Keywords:** nSTZ, type 2 diabetes, syringic acid, diabetic complications

## Abstract

This study investigated the effects of syringic acid (SA) on renal, cardiac, hepatic, and neuronal diabetic complications in streptozotocin-induced neonatal (nSTZ) diabetic rats. STZ (110 mg/kg i.p) was injected into Wistar rat neonates as a split dose (second and third postnatal day). Diabetes mellitus was diagnosed in adults by measuring fasting blood glucose levels, urine volume, and food and water intake. The treatment of SA (25 mg/kg, 50 mg/kg p.o) was given from the 8th to 18th postnatal week. To assess the development of diabetic complications and the effect of therapy, biochemical indicators in serum and behavioural parameters were recorded at specific intervals during the study period. SA (25 mg/kg, 50 mg/kg p.o) treatment reduced hyperglycaemia, polydipsia, polyphagia, polyuria, relative organ weight, cardiac hypertrophic indices, inflammatory markers, cell injury markers, glycated haemoglobin, histopathological score, and oxidative stress, and increased Na/K ATPase activity. These findings suggest that SA might significantly alleviate diabetic complications and/or renal, neuronal, cardiac, and hepatic damage in nSTZ diabetic rats.

## 1. Introduction

Type 2 diabetes mellitus (T2DM) is a non-infectious metabolic disease characterised by severe and persistent hyperglycaemia owing to decreased insulin secretion and increased insulin resistance. It is a leading global cause of death. The proportion of diabetic patients is increasing in most countries, with 374 million people at an elevated risk of developing diabetes mellitus [1]. Poor control of hyperglycaemia in diabetic patients frequently results in serious microvascular (diabetic nephropathy, neuropathy, and cardiomyopathy) and macrovascular (peripheral vascular disease and cerebrovascular disease) complications [2,3,4]. According to Ghule et al. (2012) and Shang et al. (2013), diabetic nephropathy, which manifests as microalbuminuria, impaired urinary creatinine clearance rate, renal hypertrophy, and glomerular sclerosis, is the most prevalent cause of end-stage renal disease worldwide [5,6]. Painful diabetic neuropathy affects all types of peripheral nerves, impacting practically all systems and organs in the body, including the long somatosensory nerves in the extremities, where it frequently causes sensory loss or excruciating discomfort [7]. Additionally, diabetic patients experience heart dysfunction at a rate of 2 to 5 times higher than the non-diabetics [8]. Several structural anomalies that results in left ventricular hypertrophy (LVH), systolic and diastolic dysfunction, or a combination of these, are cardinal signs of diabetic cardiomyopathy [9]. Furthermore, hyperglycaemia causes liver impairment, a common secondary diabetic complication [10,11]. Cirrhosis and hepatocellular carcinoma are among the primary causes of death in diabetic patients [12]. Ling et al., 2020 collected death certificate data for the last 17 years (2000 to 2016) from 108 countries through the WHO mortality database and concluded that deaths from diabetic vascular complications have significantly increased between 2000 and 2016 [13].

Despite the fact that there are many antihyperglycaemic medications on the market today, many of them only partially satisfy patients’ needs and have a lot of negative side effects, including a significant chance of eventual failure, lactic acidosis, liver issues, and diarrhoea [14,15]. Another problem is that after a few years of treatment, all glucose-lowering drugs may start to lose their effectiveness [16]. Therefore, a top research priority has been to discover natural phytochemicals that maintain therapeutic efficacy while having fewer negative side effects [17]. Such scientifically validated phytochemicals may be used either alone or in combination with well-established antihyperglycaemic drugs. This combination may help to lower the dose, prevent dose-related undesirable side effects, and delay the onset of tolerance to currently available antihyperglycaemic drugs. With this background knowledge, the decision was made to investigate the efficacy of natural phytochemicals against chronic diabetes complications.

**Figure 1:**
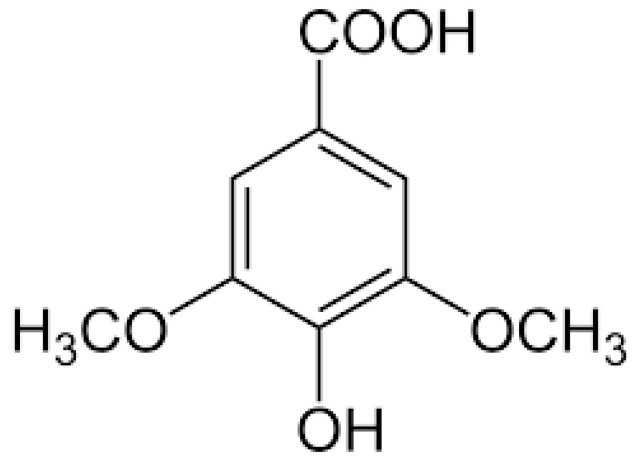
Structure of syringic acid.

SA (Figure 1) is a phenolic acid that is found in grapes, red wine, honey, acai palms, pumpkin, a variety of dry fruits (including olives and dates), spices, and other plants [18]. SA has been reported for its antidiabetic [19,20,21], antiglycating [22], antisteatosis and anti-inflammatory [23,24,25], antioxidant and antihypertensive [24,26,27], and antibacterial and antimicrobial properties [28,29]. It also has neuroprotective [30,31,32,33,34,35,36] and hepatoprotective properties [37,38], and ameliorates diabetic cataracts by suppressing aldose reductase enzyme [39]. It also has anticancer properties [40,41], is used to make dental cement [42], reduces acute thromboembolism and clot formation in mice [43], and increases gastric acid secretion [44]. It inhibits type 1 collagen formation in in vitro studies [45]. During a subacute toxicity study, SA was shown to be safe [46].

Hyperglycaemia-induced oxidative stress and the glycosylation of essential nucleic acids, lipids, and proteins may make the tissue more sensitive to oxidative damage and result in structural and functional damage to the renal glomerulus, sciatic nerve, cardiomyocytes, and hepatocytes, and finally result in the development of diabetic complications [3,4]. STZ induces oxidative stress in preclinical animal models, which causes organ damage and/or the onset and development of diabetes complications. The nSTZ model is a trustworthy, reproducible experimental animal model for T2DM and can be used to induce diabetic complications such as nephropathy, neuropathy, cardiomyopathy, and liver disease, among others [47,48]. Several natural and synthetic agents have been tested for their efficacy [47].

As per the available literature information, SA has never been studied in vivo for its effects on nSTZ-induced T2DM-associated complications (nephropathy, neuropathy, cardiomyopathy, and liver disease) and/or organ damage. Therefore, the current investigation was carried out to assess SA’s effectiveness against diabetes complications and/or organ damage caused by significant chronic hyperglycaemia, oxidative stress, and inflammation in an nSTZ animal model.

## 2. Material and Methods

### 2.1. Agents and Chemicals

Syringic acid (HSN-98020000) was procured from TCI Chemicals (Tokyo, Kanto, Japan) with 97% purity. All of the needed biochemical kits were acquired from Lab-Care Diagnostics Pvt Ltd, Mumbai, Maharashtra, India., Transasia Bio-Medicals Ltd, Mumbai, Maharashtra, India. and Tulip Diagnostics Pvt Ltd, Panji, Goa, India. Metformin (MET) was received as a gift sample from Alkem Laboratories Limited, Mumbai, Maharashtra, India. Reduced glutathione (GSH), superoxide dismutase (SOD), malondialdehyde (MDA) and streptozotocin (STZ) were procured from Sigma Chemical Co., St. Louis, Missouri, USA, and the other required chemicals utilised were of the highest quality and procured from S.D. Fine Chemicals, Mumbai, Maharashtra, India and LobaChemi Pvt. Ltd., Mumbai, Maharashtra, India.

### 2.2. Animals and Induction of T2DM-Associated Complications

Healthy adult male and female Wistar rats (150–200 g) were procured from the National Institute of Biosciences, Pune (India). They were housed at 22 ± 1°C, with a relative humidity of 45–55% under a 12 h light: 12 h dark cycle. The rats had unlimited access to Nutrivet Life Science’s regular pellet chow from Pune, India (4.3% fibre, 20% protein, 4% fat, and 57.5% carbohydrate). In our lab, a dose of STZ (110 mg/kg, i.p in split form) was established to achieve a fasting blood glucose level (FBG) of ≥150 mg/kg in adult rats. STZ (55 mg/kg, i.p.) was administered to neonates (8–10 gm) on the 2nd and 3rd postnatal days. The split dose reduces the mortality rate and causes desirable hyperglycaemia to develop diabetic complications [5,49,50,51]. In non-diabetic control groups, rat neonates were given just citrate buffer (0.1 M, pH 4.5, i.p). After 4 weeks, young rats were monitored carefully and it was ensured that they could access and/or consume food and water on their own, and then they were separated from their mothers. Animals were tested for FBG, polyuria, polyphagia, and polydipsia to diagnose diabetes after 8 weeks of age. The study included rats with FBG values of ≥150 mg/dl, a significant body weight (BW) reduction, polyphagia, polydipsia, and polyuria [52,53,54]. Thereafter, animals were grouped as per the experimental protocol. The health and behaviour of pregnant female rats, weaned pups, and adult rats were monitored by a veterinarian throughout the study period.

### 2.3. Experimental Design and Protocol

The female rats were divided randomly into the following groups, each consisting of six rats.

[A] Non-diabetic animals:

Group 1 (NC): nondiabetic control; and Group 2 (NC + SA): syringic acid (50 mg/kg, p.o. for 10 weeks) administered to the nondiabetic control group.

[B] Diabetic animals:

Group 3 (DC): n-STZ diabetic control; Group 4 (DC + SA25): syringic acid (25 mg/kg, p.o. for 10 weeks); Group 5 (DC + SA50): syringic acid (50 mg/kg, p.o. for 10 weeks); and Group 6 (DC + MET): metformin (200 mg/kg/day p.o. for 10 weeks).

Doses of SA were selected based on the therapeutic efficiencies reported in previous research reports [19,20,21,37,55,56] and, in addition, our previous studies suggested that the LD_50_ of SA is greater than 2000 mg/kg [46], which is in concordance with the results of Pawar et al., 2021 [21].

### 2.4. Sample Collection and Tissue Preparation

At the end of the 18th week, behavioural and physical parameters were assessed; animals were anaesthetised with isoflurane (Abbott Pharma Pvt Ltd., India) and blood samples were collected and centrifuged at 5000 rpm for 10 min (Remi CM 12, Remi ElectroTechnik Ltd.) after blood coagulation. For additional biochemical investigation, sera were collected and kept at −80 °C. A total of 0.5 mL of blood was collected in pre-calibrated tubes coated with ethylene diamine tetra-acetic acid for glycated haemoglobin (HbA1C) testing. After that, phenol red meal was given orally; 20 min later, animals were immediately sacrificed. Stomach and small intestine were removed and processed for measuring gastric emptying time (GE) and small intestine transit (IT). Body organs such as heart, kidney, and liver were isolated and weighted to measure relative organ weight. Femur length was measured. Left and right heart ventricles were weighted; left ventricle wall thickness was measured. A portion of heart, liver, right kidney, and right sciatic nerve was isolated and fixed in 10% formalin solution and stored for histopathological studies. Other portions of organs were weighted, then homogenised in buffer (pH = 7.4) and processed for the estimation of oxidative stress parameters and Na/K ATPase estimations.

### 2.5. Estimation of Biochemical and Behavioural Parameters

The development of diabetic complications has been confirmed by measuring FBG, blood urea nitrogen (BUN), serum creatinine (SCr), total cholesterol (TC), triglyceride (TG), aspartate transaminase (AST), alanine transaminase (ALT) [57,58,59,60], and behavioural markers (cold allodynia and thermal hyperalgesia) [53,61] at the 8th, 13th, and 18th week.

### 2.6. Body Weight, Urine Output, and Food and Water Intake

At the 8th and 18th postnatal week, BW, urine output (UOP), water intake (WI), and food intake (FI) were measured, and % change in BW, FI/100 gm b.w, WI/100 gm b.w, and UOP/100 gm b.w were calculated [53,62,63]. Rats were housed in metabolic cages (V.N. Shah Pvt Ltd., Mumbai, India) for 24 h to collect urine.

### 2.7. Warm and Cold Water Tail-Immersion Test

The tail-immersion test was carried out as per the method described by Necker and Hellon (1977) to determine spinal temperature sensitivity [64]. The method described by Anjaneyulu and Chopra (2004) and Attal N. et al. (1990) was used for warm and cold water tail-immersion tests to detect thermal hyperalgesia and cold allodynia in diabetic rats, respectively [65,66].

### 2.8. Measurement of Blood and Urine Biochemical Parameters

At the end of the 18th week, animals were fasted for the entire night, blood was drawn, and serum was separated. The serum biochemical parameters were estimated by by spectrophotometer (Jasco, V-730, Japan) using analytical-grade kits for BG, BUN, SCr, TC, high-density lipoprotein (HDL), TG, low-density lipoprotein (LDL), AST, ALT, total protein (TP), atherogenic index (AI)**,** albumin (ALB), C-reactive protein (CRP), lactate dehydrogenase (LDH), total bilirubin (TB), Creatinine Kinase-MB (CK-MB), and HbA1C.

The Friedewald equation was used to calculate LDL: TC-[HDL+ (TG/5)]

AI = TC-HDL cholesterol divided by HDL-cholesterol [50].

Urinary biochemical parameters such as creatinine (UCr), creatinine clearance rate (CrCl), urinary albumin (UALB), sodium (Na), and potassium (K) were qualitatively measured in 24 h urine samples using analytical-grade kits.

### 2.9. Measurement of Physical Parameters

At the end of the 18th week, rats were sacrificed. Heart, kidney, and liver were isolated. Organ weights, including that of the heart ventricle, were measured. Femur length was measured to calculate hypertrophic parameters [67,68]. The left ventricular wall thickness was measured with a calibrated vernier calliper, and the left ventricle to right ventricle weight ratio was calculated [69] along with the left ventricle hypertrophy index [54].

Furthermore, % GE was determined using the method previously described by Qiu, W.C et al., 2008 and Zheng, Q. et al., 2008 [70,71]. The % IT was determined by the following method described by Janseen and Jagenerous (1957) and Peddireddy (2010) [72,73].

### 2.10. Evaluation of Oxidative Stress Level in Renal, Cardiac, Hepatic and Neuronal Tissues

Tissue homogenates were prepared using ice-cold 0.1M phosphate buffer pH 7.4 and utilised to estimate MDA content. The supernatant was centrifuged again at 20,000 rpm for 20 min at 4 °C, and the resulting post-mitochondrial supernatant was used to calculate total protein, SOD, GSH, and NO content. Protein concentration was calculated using the method described by Lowry et al. (1951) [74]. SOD assay, GSH assay, MDA assay, and NO level were estimated as per the methods described by Misera and Fridovich (1972) [75], Moron et al. (1979) [76], Ohkawa et al. (1979) [77], and Griess (1879) [78], respectively.

### 2.11. Determination of Membrane-Bound Inorganic Phosphate Determination (Na-K ATPase)

The Na/K-ATPase activity (nmole of inorganic phosphorus/mL/1h/mg of protein) was measured as described by Svoboda and Mosinger (1981), Rao and Deshpande (2005), and Babu and Ramanathan (2011) [79,80,81].

### 2.12. Histopathological Examination

The kidney, liver, heart, and sciatic nerve tissues were fixed in 10% formalin solution, cut, and stained with haematoxylin and eosin (H and E). The histological sections were rated approximately on a scale of 0 to 4, with no change (0), minimal changes (+1), mild changes (+2), moderate changes (+3), and severe changes (+4) based on pathological alterations in the understudied tissues.

## 3. Results and Discussion

The n-STZ-induced T2DM model has been well established, thoroughly studied, and extensively reviewed as an animal model to induce diabetic complications characterised by hyperglycaemia, polydipsia, polyphagia, polyuria, insulin resistance, reduced BW, increased nociceptive threshold, elevated transaminase levels, decreased CrCl, hypertrophy (renal, cardiac, and hepatic), increased TG, TC, and UALB, reduced HDL, the death of cardiomyocytes, glomeruli, hepatic and sciatic tissue, etc. [47].

In the present study, the FBG level was significantly increased from the 8th to 18th weeks of age in the diabetic control group as compared to the nondiabetic group. Additionally, polyuria, polydipsia, and polyphagia are typical diabetes symptoms that worsen when FBG levels rise. In the present study, all the nSTZ diabetic rats showed polyuria, polydipsia, and polyphagia at the end of 8 postnatal weeks, and 10-week chronic treatment with SA (25 mg/kg, 50 mg/kg p.o) significantly lowered FI/100 gm b.w, WI/100 gm b.w, UOP/100 gm b.w. (Table 1), and FBG levels (Table 2) at the 18th week. Low levels of glucagon-like peptide-1 (GLP-1) content in the jejunum, ileum, and colon were reported in nSTZ diabetic rats [82]; GLP-1 deficiency increases appetite, cell apoptosis, hepatic gluconeogenesis, and hepatic and muscular insulin resistance while decreasing insulin secretion, cell proliferation, and satiety [83]. Hence, SA may be evaluated for GLP-1 receptor agonistic activity.

**Figure 2:**
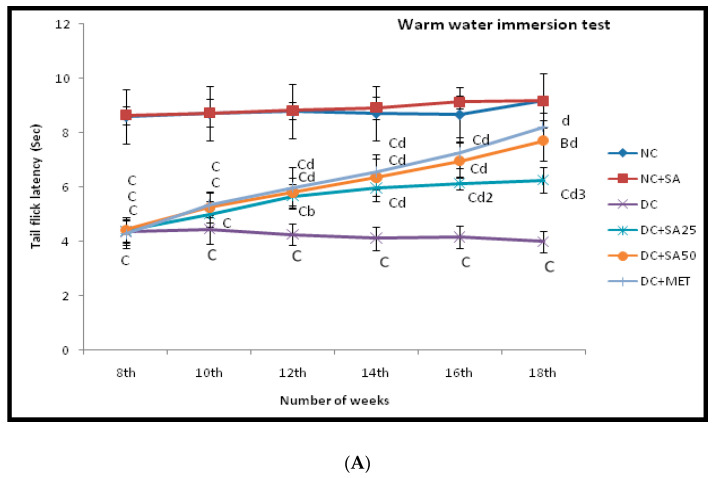

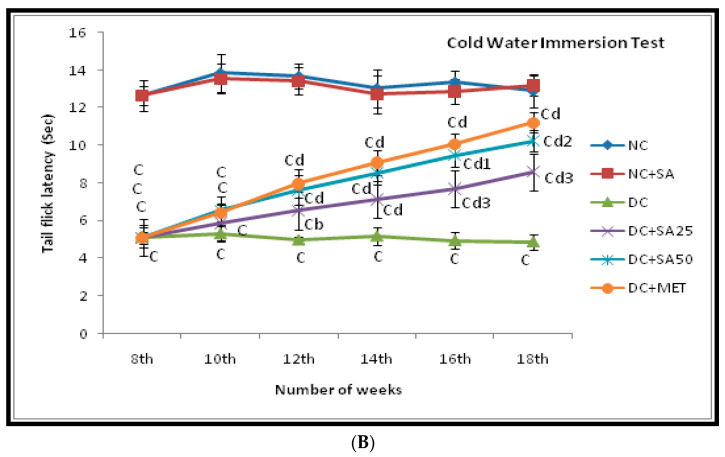
Effect of 10 weeks of repeated dose treatment of SA on (**A**) thermal hyperalgesia in hot water tail immersion test and (**B**) cold allodynia in cold water immersion test.

By the end of the 8th week, a significant increase in all crucial in-life biochemical evaluation parameters (FBG, SCR, BUN, TG, TC, ALT, and AST) (Table 2) and behavioural markers (Figure 2) was observed in all nSTZ diabetic rats as compared to the nondiabetic group. Furthermore, a significant enhancement in all these parameters was observed at the end of the 13th and 18th weeks. Treatment with SA (25 mg/dL, 50 mg/kg p.o) and MET (200 mg/kg p.o) reduced the raised level in an efficient and dose-dependent manner as compared to nSTZ diabetic groups; hence, it is evident that the diabetic complications progressively develop in the nSTZ diabetic rats and treatment with SA and MET has a positive effect against them.

Excessive tissue protein breakdown is responsible for a lower BW. The administration of SA (25 mg/kg, 50 mg/kg p.o) to nSTZ diabetic rats resulted in a significant increase in BW, indicating that muscle tissue damage caused by hyperglycaemia was prevented. The antidiabetic effects of SA in the present study are in agreement with the findings of previous research reports based on a type 1 diabetes mellitus (T1DM) animal model [19,20,21]. Furthermore, uncontrolled hyperglycaemia promotes the generation of excessive free radicals, reducing the synthesis of SOD and GSH, which exaggerate a weakened defence system and tissue injury [84]. SA was discovered to be a powerful antioxidant and its ability to scavenge free radicals may be responsible for its hypoglycaemic activity [24,26,27].

In the present study, nSTZ diabetic rats demonstrated a pathological rise in BUN, SCr, UALB, and dyslipidaemia, and decreased CrCl at the age of 18 postnatal weeks (Table 3 and Table 4). These symptoms are indicators of diabetic nephropathy [47,63]. Chronic treatment of SA (25 mg/kg and 50 mg/kg p.o) significantly restored the levels of BUN, SCr, and urinary ALB, as well as dyslipidaemia and CrCl (Table 3 and Table 4). SA’s antioxidant, anti-inflammatory, and antihyperglycaemic properties could be the cause of its positive effects, which are in concurrence with previous research reports on SA in other animal models [20,55].

UCr: urinary creatinine; CrCl: creatinine clearance rate; UALB: urinary albumin; Na: sodium; K: potassium; NC: nondiabetic control; NC + SA: syringic acid (50 mg/kg, p.o. for 10 weeks) administered to nondiabetic control group; DC: n-STZ diabetic control; DC + SA25: syringic acid (25 mg/kg, p.o. for 10 weeks) administered to diabetic animals; DC + SA50: syringic acid (50 mg/kg, p.o. for 10 weeks) administered to diabetic animals; and DC + MET: metformin (200 mg/kg/day p.o. for 10 weeks) administered to diabetic animals.

nSTZ diabetic rats showed an increase in AST, ALT, and BR levels (Table 4), which is consistent with research conducted on nSTZ diabetic rats by Abdollah, M. et al. (2010) and Shinde and Goyal (2003) [85,86], and a high level of TB was observed in the STZ-induced T1DM rat model [87]. In contrast, the SA (25 mg/kg and 50 mg/kg p.o) and MET (200 mg/kg p.o) treatment of nSTZ diabetic rats reduced the AST, ALT, and TB levels (Table 4), indicating a protective role against liver dysfunction. In other rat models, Srinivasan et al., 2014, Sammeturi et al., 2020, Sabahi et al., 2020, and Ramachandran and Raja, 2010, reported similar effects of SA [20,37,55,56]. Mohamed et al. (2016) suggested that insulin resistance accompanied by oxidative stress and aberrant inflammatory signals could contribute to liver damage [60]. Hence, the antidiabetic, anti-inflammatory, and antioxidative properties of SA might be the main cause of its protective effect on diabetes-induced liver damage.

According to reports, TGF-1 decreases reabsorption in proximal tubes and increases glomerular permeability to cause albuminuria [88]. Additionally, serum ALB has a significant direct association with CrCl and an indirect association with UALB level in diabetic patients with nephropathy [89,90]. In our study, a reduced level of serum ALB was observed in nSTZ diabetic rats (Table 4); this concurs with research conducted in the T1DM animal model by Baig et al. (2011) and Sundaram, E.N et al. (2009) [63,91]. SA (25 mg/kg and 50 mg/kg p.o) and MET (200 mg/kg p.o) treatment reversed the decline in serum ALB, which is in accordance with other findings of the current study, such as increased CrCl and a decrease in UALB (Table 4). Sinapic acid was found to decrease TGF-β1 level in rats [92]. SA, a sinapic acid derivative, could be acting as an inhibitor of TGF-β1.

Glial cell dysregulation and the demyelination of nerves are caused by hyperglycaemia and oxidative stress, resulting in a decrease in nerve conduction velocity (NCV) [93]. In the present study, nSTZ diabetic rats showed reduced tail withdrawal latency while exhibiting cold allodynia and thermal hyperalgesia (Figure 2A,B); this is consistent with the results of other studies using rat models for STZ-induced T1DM and nSTZ-induced T2DM [54,61,65,94]. SA (25 mg/kg and 50 mg/kg p.o) and MET (200 mg/kg p.o) treatment significantly increased the tail withdrawal latency (Figure 2A,B). According to previous research reports, SA reduced the inflammation and demyelination in the sciatic nerve [32]. Dyslipidaemia is a novel target in the treatment of diabetic neuropathy [95]. In the present study, SA (25 mg/kg and 50 mg/kg p.o) treatment improved dyslipidaemia. These findings support the beneficial effect of SA in the treatment of diabetic neuropathy. Delayed gastrointestinal transit is a well-known diabetic complication; its pathogenesis could be linked to disturbances in gastric motor activity [96]. Reduced gastrointestinal motility has also been reported in diabetic patients [97] as well as nSTZ-induced T2DM rat model [98,99]. In the present study, nSTZ diabetic rats showed delayed %GE and %SIT; in contrast, chronic treatment of SA and MET increased the %GE and %SIT (Table 5). SA’s anti-inflammatory effect via the inhibition of inflammatory kinase c-jun N-terminal kinase (JNK) and its ability to reduce demyelination and improve dyslipidaemia may explain the mechanisms behind its beneficial effect [100]. SA’s neuroprotective effect is consistent with the findings of Tokmak et al., 2015, Pawar et al., 2021, and Cao et al., 2016 [21,30,35].

Insulin stimulates lipoprotein lipase, which hydrolyses TG [101]. Insulin deficiency causes hypertriglyceridemia. By stimulating the inflammatory cascade, including oxidative stress, Rho-kinase has a substantial impact on the pathogenesis of diabetic nephropathy [102]. Serum TC, TG, and protein levels all dropped significantly when BG was normalised [103]. In the present study, urinary ALB, serum TC, and TG levels were greatly elevated in n-STZ-treated rats, and chronic SA treatment significantly restored these elevated levels (Table 3 and Table 4); SA may be working as a Rho-kinase inhibitor and lipoprotein lipase activator in this case. Similar effects of SA in other animal models were reported by Srinivasan et al., 2014, and Sammeturi et al., 2020 [20,56]. Myocardial degeneration, pyknotic nuclei, elevated serum CK-MB, and LDH levels are signs of cardiomyocyte damage brought on by hyperglycaemia and dyslipidaemia [104,105]. Reduced LDH and CK-MB levels in SA-treated nSTZ diabetic rats support its lipid-lowering effect in the prevention of hyperglycaemia/hyperlipidaemia-induced myocardial injury (Table 4).

**Figure 3:**
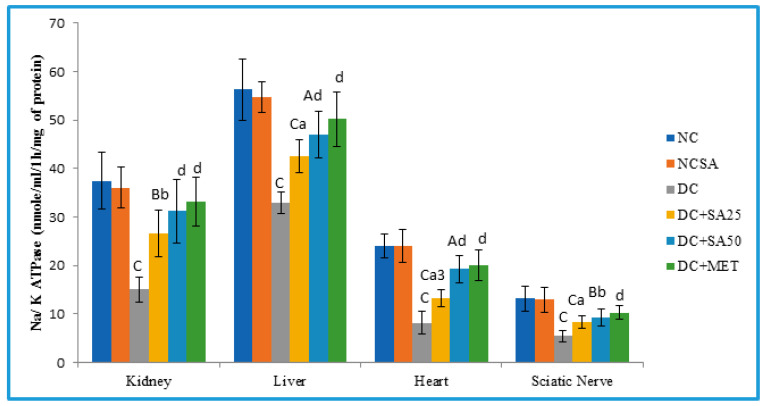
Effect of 10 weeks of repeated dose treatment of SA on Na/K ATPase level in kidney, liver, heart, and sciatic nerve of rats with nSTZ-induced T2DM at the end of the 18th week. Data are expressed as mean ± SD, where *n* = 6 rats per group; A (*p*< 0.05), B (*p*< 0.01), and C (*p* < 0.001) indicate a significant difference from the normal control; a (*p*< 0.05), b (*p*< 0.01), and d (*p* < 0.001) indicate a significant difference from the diabetic control; and 1 (*p*< 0.05), 2 (*p*< 0.01), and 3 (*p* < 0.001) indicate a significant difference from the metformin-treated group.

The Na/K ATPase creates and maintains trans-sarcolemmal Na and K gradients in cardiac cells, responsible for the secondary active transport of bile acids and Na across the plasma membrane of hepatic cells [106,107]. It plays a key role in membrane depolarisation and regulates NCV. Decreased Na/K ATPase activity reduced ATP hydrolysis and decreased Na reabsorption, leading to increased diuresis [108]. Additionally, diabetic cardiomyopathy [109], nephropathy [110], neuropathy [53], and diabetic liver [111] have all been linked to decreased Na/K ATPase activity. Therefore, it is essential to determine the Na/K ATPase level to investigate the protective effect of SA on the progression of diabetic complications. nSTZ diabetic rats showed decreased Na/K ATPase activity in all understudied body organ homogenates (Figure 3). The cause could be oxidative stress, which causes thiol group oxidation in the Na/K ATPase [112], excessive nonenzymatic glycation of the enzyme, membrane fluidity loss [108], and AMPK/SIRT1 pathway confounding by advanced glycated end product (AGE). The reduced membrane-bound Na/K ATPase activities were alleviated by treatment with SA (Figure 3), which could be due to its antioxidative properties or its ability to conserve the integrity of cell membranes. This finding is associated with a significant decline in Na/K ATPase activity and a corresponding drop in urine volume, as well as cold allodynia and thermal hyperalgesia in SA treatment groups. Additionally, in our experiments, we observed increased urinary Na^+^ and reduced K^+^ excretion in nSTZ rats, which was significantly reversed by SA treatment; the reason could be the improvement in the activity of Na/K ATPase by SA.

AGEs result in the production of free radicals, extracellular matrix build-up, mesangial cell hypertrophy, endothelial damage, sensory nerve damage, a decrease in the viability of rat Schwann cells, systolic and diastolic dysfunction, and inflammation in hepatocytes and hepatic stellate cells; all these changes finally lead to diabetic nephropathy, neuropathy, cardiomyopathy, and liver injury [113,114,115]. Furthermore, AGE has been found in the peripheral nerves of diabetic patients [116]. The amount of HbA1c was regarded to be a critical indicator of AGEs and an in vitro investigation revealed that oxidative stress increases the glycation of Na/K ATPase (when incubated with glucose), diminishing its function [117]. Chronic treatment with SA and MET considerably suppressed this high amount of HbA1c (Table 4) and increased the Na/K ATPase pump’s activity; this effect of SA is in agreement with the findings of previous research reports on SA in the T1DM animal model [19,20]. SA may reduce the accumulation of AGE in understudied tissues and AGE’s interaction with RAGE, which causes an increase in oxidative stress and the inflammatory response [114,115].

**Figure 4:**
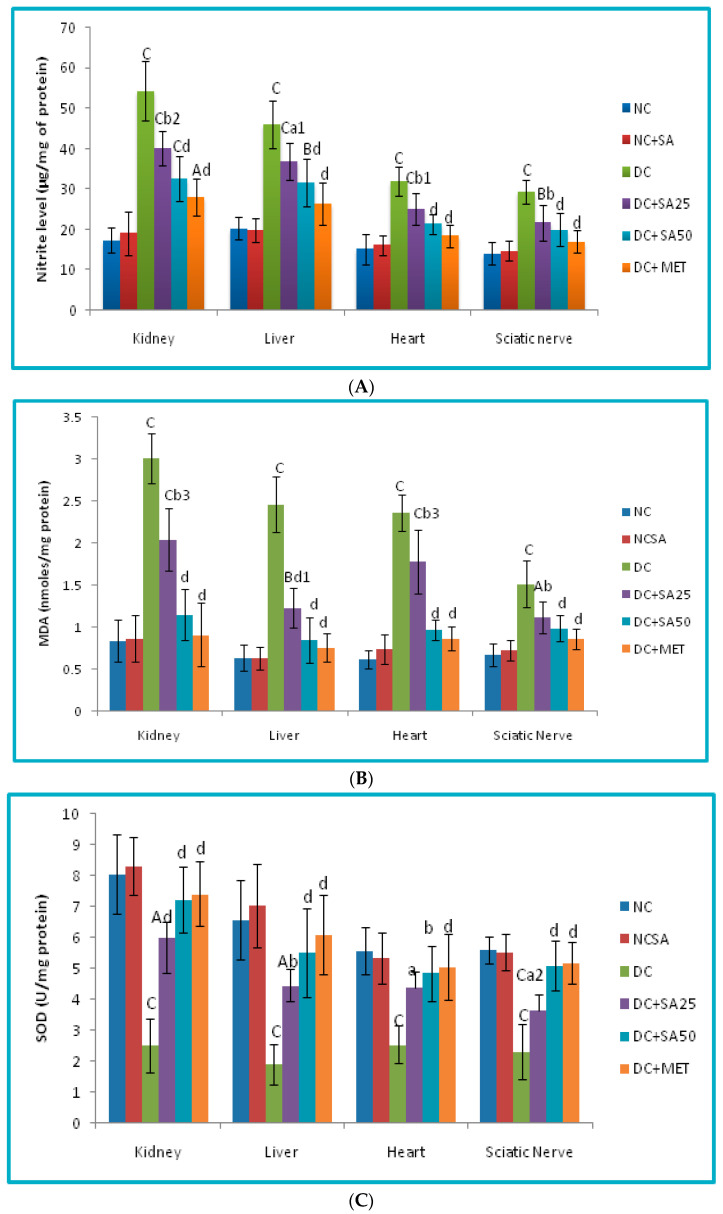

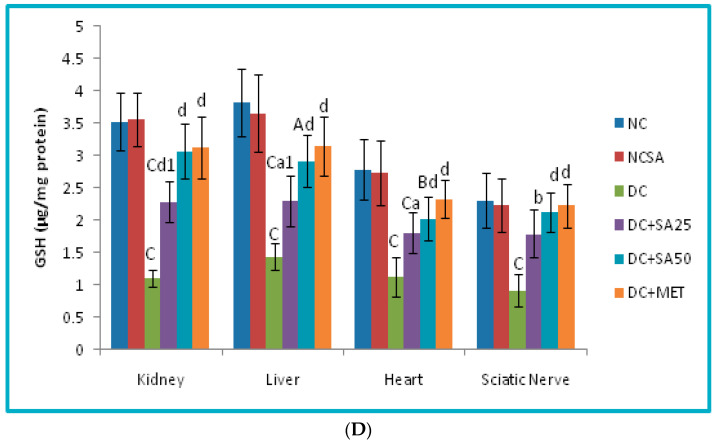
Effect of 10 weeks of repeated dose treatment of SA on (**A**) nitrite level, (**B**) MDA level, (**C**) SOD level, and (**D**) GSH level in kidney, liver, heart, and sciatic nerve of rats with nSTZ-induced T2DM at the end of the 18th week. Data are expressed as mean ± SD, where *n* = 6 rats per group; A (*p* < 0.05), B (*p* < 0.01), and C (*p* < 0.001) indicate a significant difference from the normal control; a (*p* < 0.05), b (*p* < 0.01), and d (*p* < 0.001) indicate a significant difference from the diabetic control; and 1 (*p* < 0.05), 2 (*p* < 0.01), and 3 (*p* < 0.001) indicate a significant difference from the metformin-treated group.

High MDA content, low SOD activity, and a low GSH level have been reported in patients with diabetic complications [118]. In the present investigation, high MDA content, low SOD, and a low GSH level were observed in all understudied tissues of nSTZ diabetic rats (Figure 4). However, SA and MET treatment significantly restored MDA content, SOD activity, and GSH level. These effects of SA are in agreement with the findings of previous research reports in other animal models [30,119]. SA might inhibit apoptosis, oxidative DNA damage, and neuronal pain, and improve the NCV, myelination of the sciatic nerve, glomerular injury, and liver function via restoring MDA content, SOD, and the GSH level [30,100,119,120,121,122,123]. Therefore, SA could be considered a component of a therapeutic regimen for delaying diabetic complications.

High levels of NO have also been observed in diabetic patients and diabetic animal models [124,125]. NO increases lipid peroxidation and the nitration of protein, and affects the signal transduction pathways in nerve cells, causing endoneurial hypoxia, which leads to neuropathy [93,126]. The increased NO generation could be a factor in the UALB and hyperfiltration that define diabetic nephropathy [127], as well as impair cardiovascular function [128]. The increased activity and expression of inducible nitric oxide synthase (iNOS) were found in the liver and hepatocytes of STZ-treated rats [129]. This could be a consequence of decreased PI3K levels; insulin was found to be an activator of PI3K [130]. In the present study, SA reduced the NO level in the liver. The reason could be the activation of PI3K followed by a decline in the expression and activity of iNOS. When free radicals are produced as a result of prolonged hyperglycaemia, nitrosative stress is unavoidably increased. The increased level of NO in our findings demonstrates the same (Figure 4). The present effect of SA is consistent with that found by previous researchers [131,132].

In our study, there was an elevated serum CRP level in nSTZ diabetic rats, which is consistent with earlier studies demonstrating increased serum CRP levels in diabetic individuals and rats with STZ-induced T2DM [133,134]. Diabetic rats given SA had considerably lower CRP levels in their blood (Table 4). As a result, SA’s positive effects are linked to its ability to reduce inflammation. The CRP-reducing effect of SA is in accordance with the findings of Sammeturi et al. (2020), where SA attenuated inflammation in rats treated with isoproterenol to induce post-myocardial toxicity [56].

**Figure 5:**
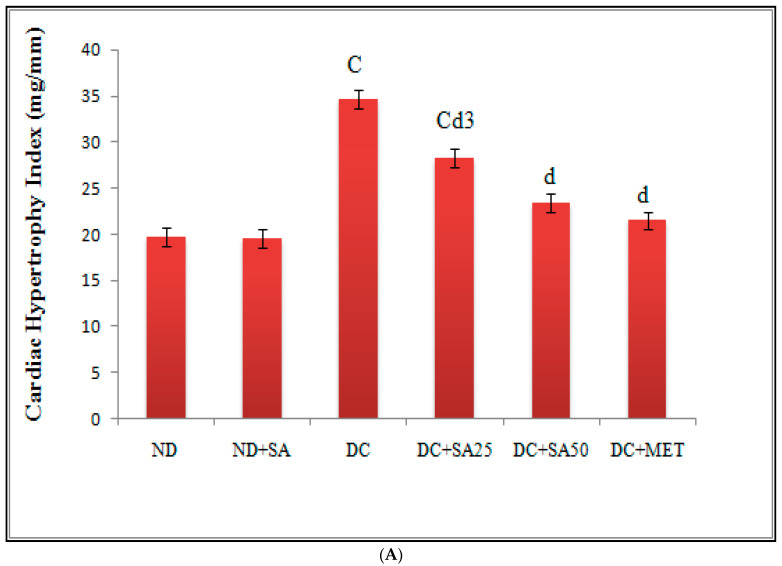

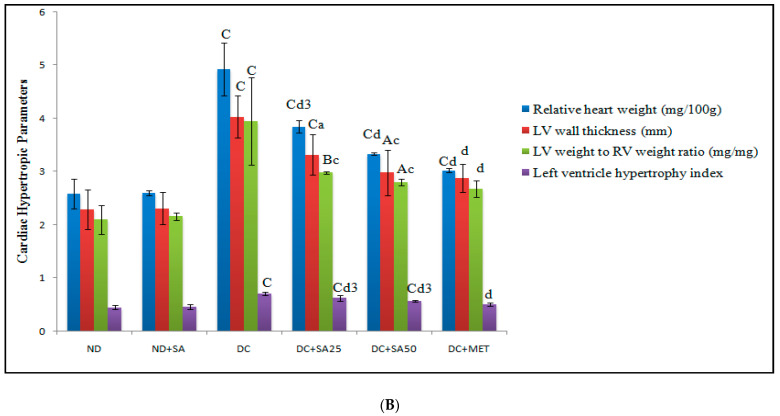
Effect of 10 weeks of repeated dose treatment of SA on (**A**) cardiac hypertrophic index, and (**B**) cardiac hypertrophic parameters in rats with nSTZ-induced T2DM at the end of the 18th week. Data are expressed as mean ± SD, where *n* = 6 rats per group; A (*p* < 0.05), B (*p* < 0.01), and C (*p* < 0.001) indicate a significant difference from the normal control; a (*p* < 0.05), b (*p* < 0.01), and d (*p* < 0.001) indicate a significant difference from the diabetic control; and 1 (*p* < 0.05), 2 (*p* < 0.01), and 3 (*p* < 0.001) indicate a significant difference from the metformin-treated group.

In hyperglycaemic circumstances, oxidative stress and inflammation are known to cause cardiomyocyte, renal, and hepatic hypertrophy [135,136]. Here, nSTZ diabetic rats exhibited cardiac hypertrophy, evidenced by the increased relative cardiac weight, cardiac hypertrophy index, left ventricular hypertrophy index, ratio of left ventricular weight to right ventricular weight, and left ventricular wall thickness (Figure 5A,B). Furthermore, increases in relative renal and hepatic weights were observed in nSTZ diabetic rats (Table 3). In the nSTZ diabetic rat, similar findings on cardiac [54], hepatic [137], and renal hypertrophy [5] were reported. Chronic SA therapy reduced cardiac hypertrophy; this benefit could be linked to the diabetic myocardium’s higher rate of fatty acid oxidation, which reduces inflammation, oxidative stress, and lipid build-up [138]. Furthermore, a decline in renal relative weight could be related to a decrease in glomerular basement thickness, which would limit glomerular hypertrophy [63]. SA treatment decreases relative hepatic weight; the reason might be decreased TG accumulation, which could be due to the increased influx of fatty acids into the liver induced by hepatic insulin resistance [136]. Consistent with our findings, SA has prevented isoproterenol-induced cardiac hypertrophy in rats [56]. No reports currently exist on the effect of SA on diabetes-induced renal and hepatic hypertrophy, but the parent group sinapic acid reversed renal hypertrophy in T1DM [139] and reduced relative hepatic weight in rats with chronic alcohol-induced liver injury and steatosis [140]. Thus, the mechanism of reducing hypertrophy could result in an improvement in dyslipidaemia, TGF-β1 inhibition, and anti-inflammatory and antioxidative effects.

SA has a hydroxyl group at the C4 position and two methoxy groups at the C3 and C5 positions (Figure 1). The number of hydroxyl and methoxy moieties determines antiradical strength [141,142]. The phenolic hydroxyl group and CH = CHCOOH moiety encourage the antioxidant property of phenolic acids when the other substituents on the benzene ring are the same, which acts as an electron donor [143,144]. Therefore, the protective effect of SA in diabetic complications through its antioxidant potential may be ascribed to its structural properties.

**Figure 6:**
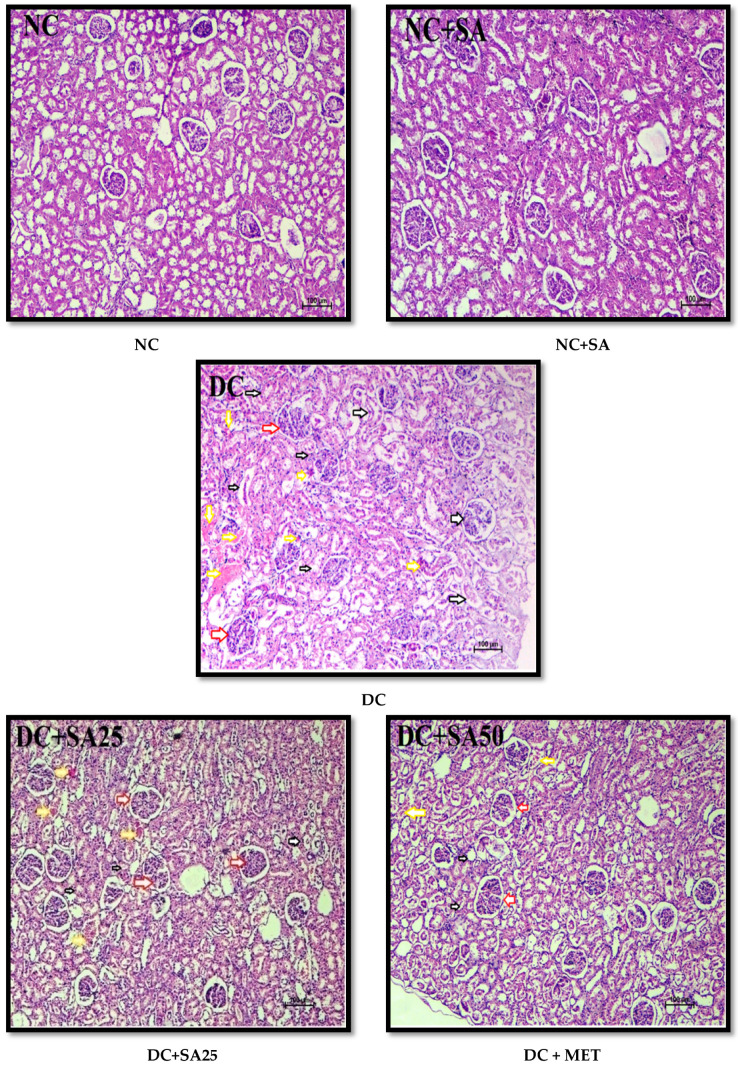

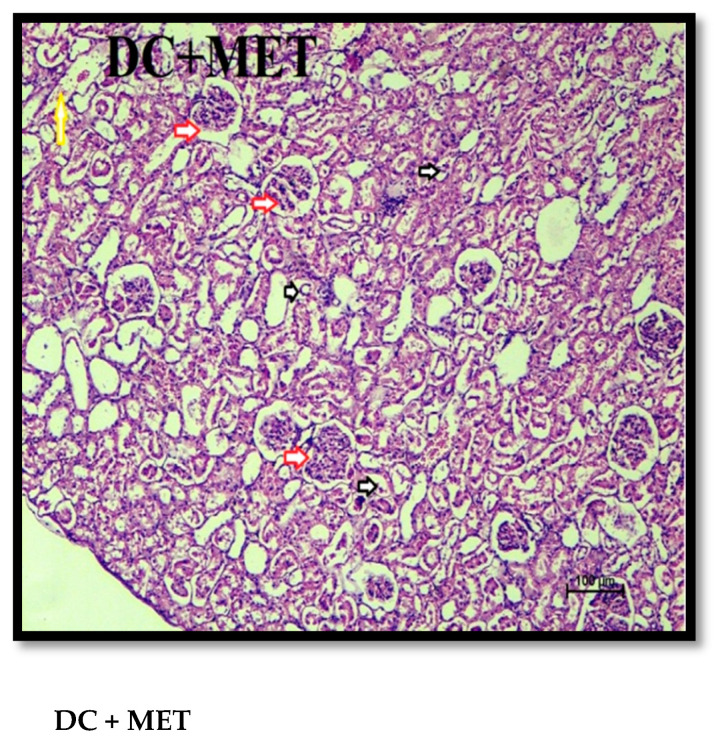
Effect of 10 weeks of repeated dose treatment of SA on the histopathological characteristics of the kidney of rats with nSTZ-induced T2DM at the end of the 18th week. Glomerular damage (red arrow), haemorrhage (yellow arrow), and tubular damage (black arrow).

**Figure 7:**
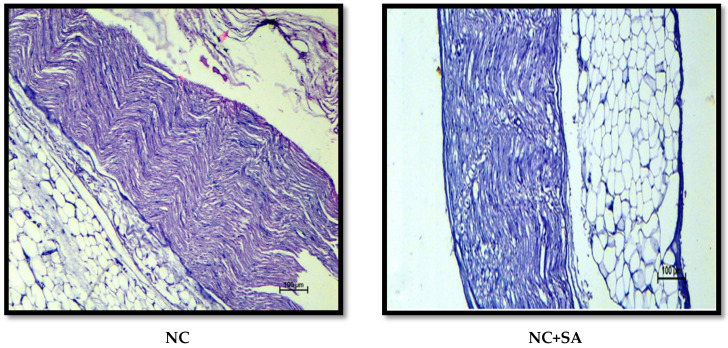

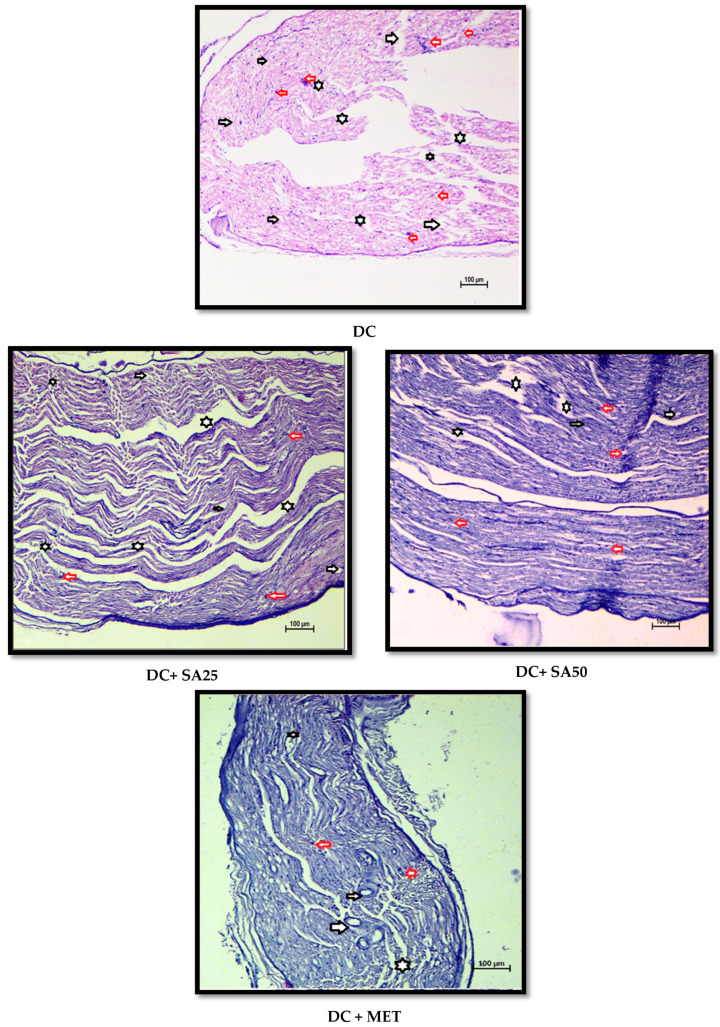
Effect of 10 weeks of repeated dose treatment of SA on the histopathological characteristics of the sciatic nerve at the end of 18th week. Nerve cell vacuolation (black arrow), necrotic changes (black star), and mononuclear cell infiltration (red arrow).

**Figure 8:**
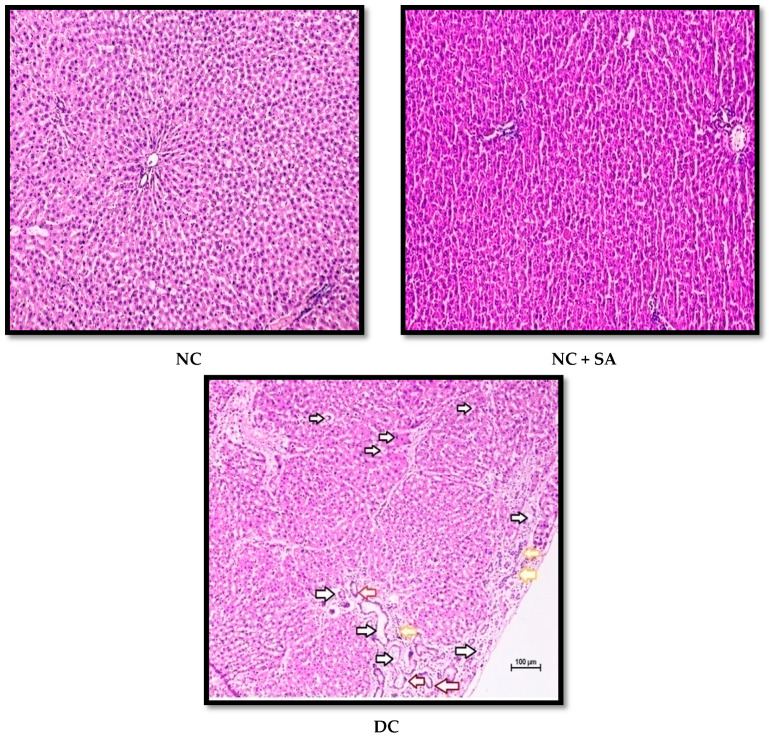

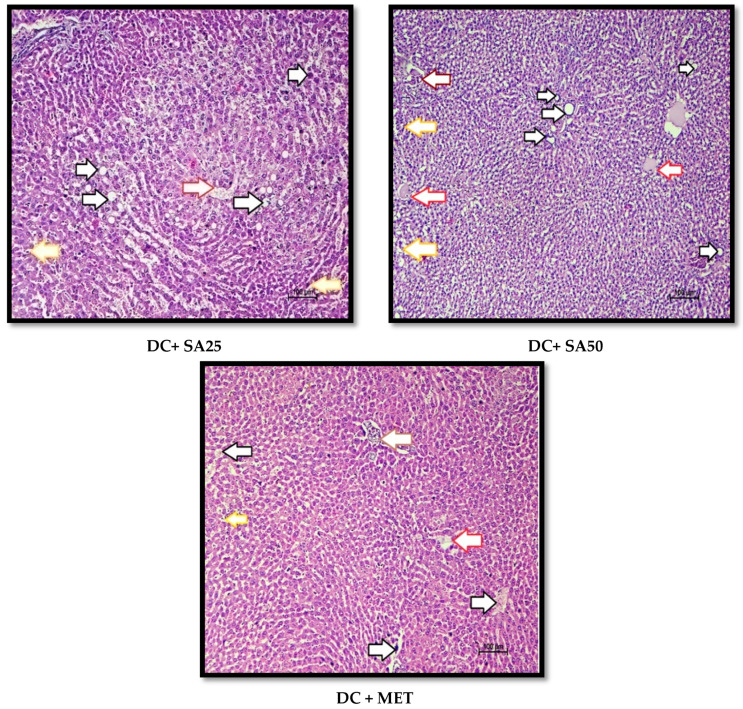
Effect of 10 weeks of repeated dose treatment of SA on the histopathological characteristics of the liver of rats with nSTZ-induced T2DM at the end of the 18th week. Degenerative changes in hepatocytes (black arrow), focal congestion of central vein (red arrow), mononuclear cell infiltration (yellow arrow), and necrosis of the portal tract (maroon arrow).

**Figure 9:**
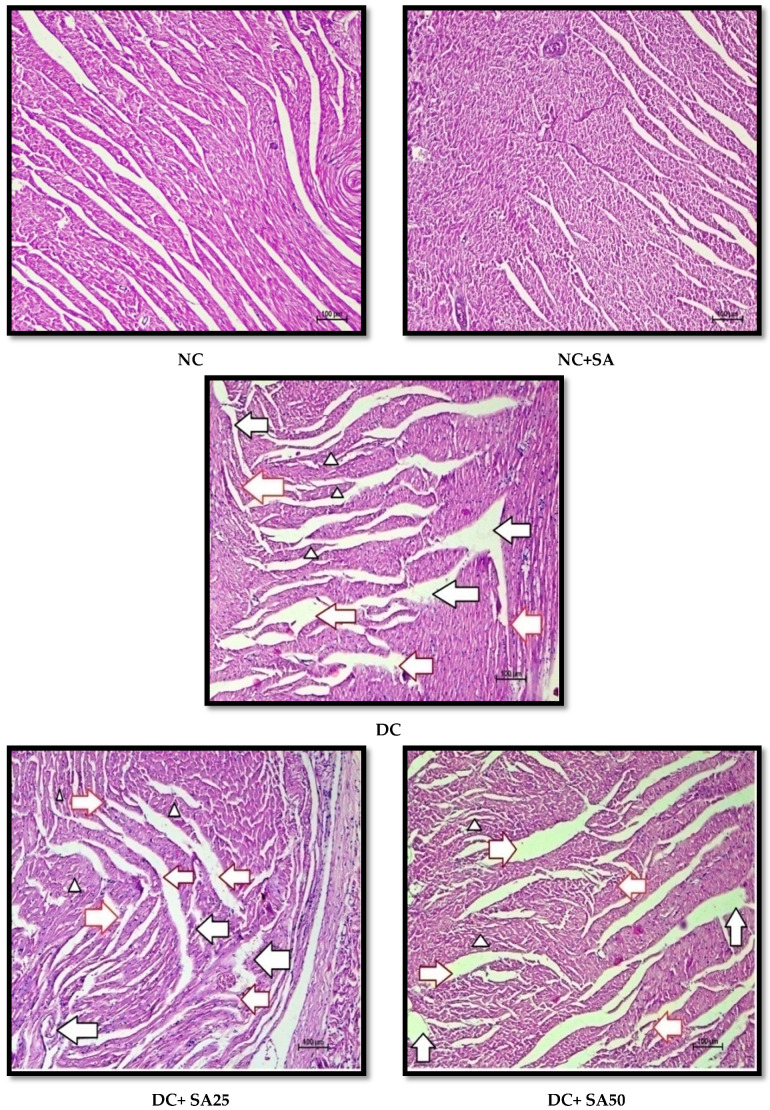

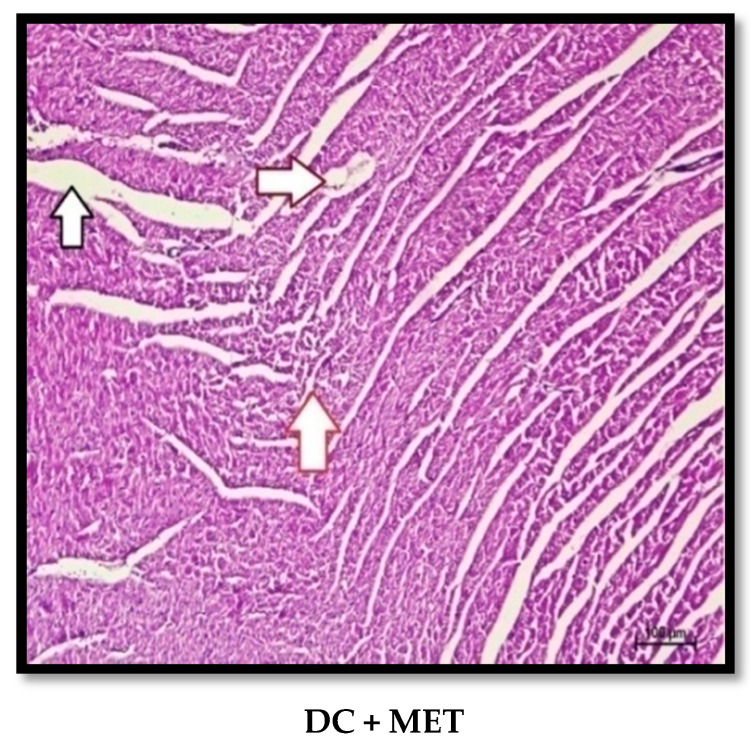
Effect of 10 weeks of repeated dose treatment of SA on the histopathological characteristics of the heart of rats with nSTZ-induced T2DM at the end of the 18th week.Ruptured muscle fibre (black arrow), cellular disarray (red arrow), atropic muscle fibres (triangle), and reduced extracellular space (maroon arrow).

**Figure 10:**
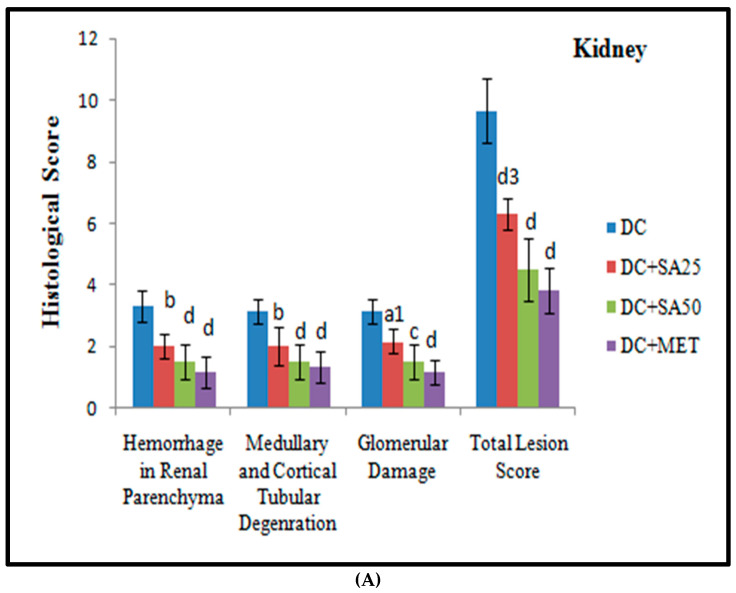

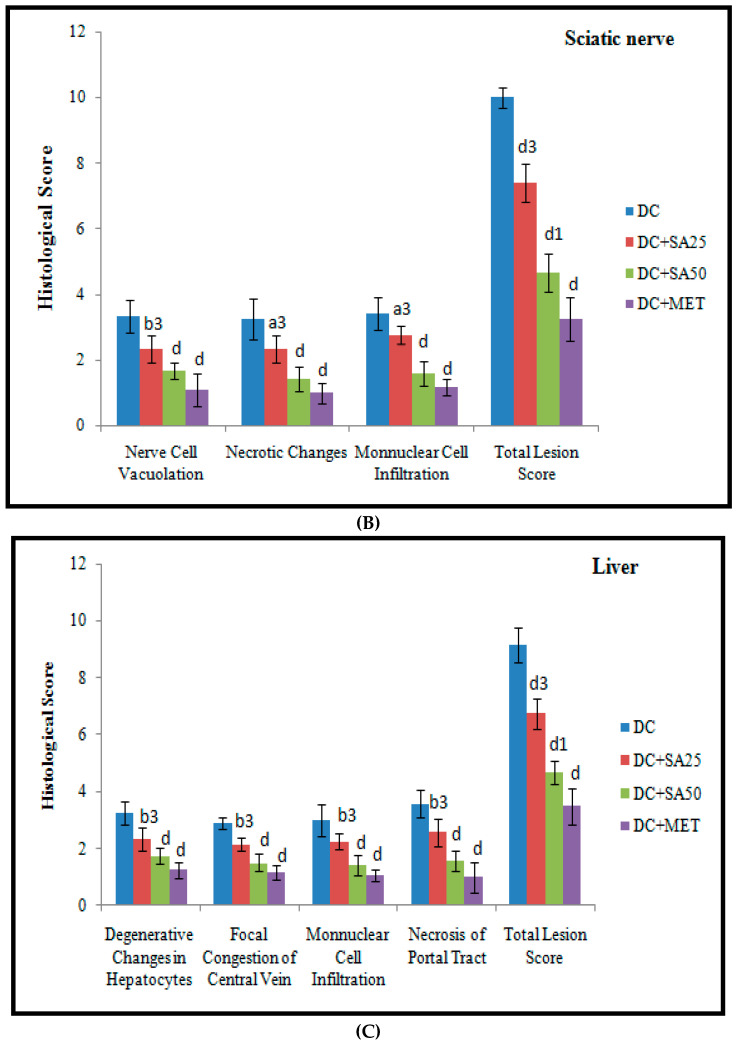

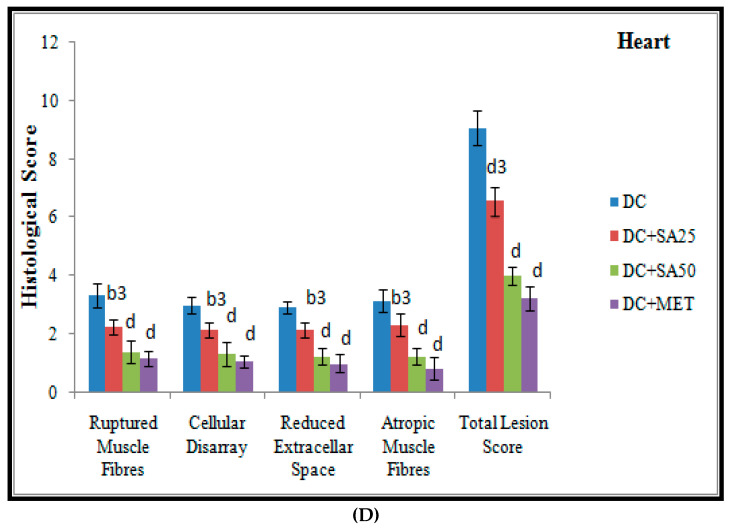
Graphical representation of histopathology scores for (**A**) kidney, (**B**) sciatic nerve, (**C**) liver, and (**D**) heart of rats with nSTZ-induced T2DM at the end of the 18th week. Data are expressed as mean ± SD from six rats and were analysed by two-way ANOVA followed by Tukey’s test. a (*p* < 0.05), b (*p*< 0.01), and d (*p* < 0.001) indicate a significant difference from the diabetic control; and 1 (*p* < 0.05), 2 (*p* < 0.01), and 3 (*p* < 0.001) indicate a significant difference from the metformin-treated group.

Chronic hyperglycaemia in STZ diabetic rats causes significant damage to the kidney, sciatic nerve, heart, and liver structures; these changes are possibly associated with the generation of free radicals [63,145,146]. The H and E-stained renal, cardiac, liver, and sciatic nerve tissue sections revealed normal tissue structure in nondiabetic animals. nSTZ diabetic rats had significantly higher histological scores for renal damage (*renal tissue haemorrhage in parenchyma, medullary and cortical tubular degeneration*, and *glomerular damage)*, sciatic nerve tissue damage (*nerve cell vacuolation, necrotic changes, and mononuclear cell infiltration*), liver damage (*degenerative changes in hepatocytes*, *focal congestion of the central vein, mononuclear cell infiltration, and necrosis of the portal tract)*, and cardiac damage (*ruptured muscle fibres, cellular disarray, reduced extracellular space, and atropic muscle fibres*) when compared with the nondiabetic group. SA and MET treatment protect against these histopathological changes by increasing antioxidant enzyme levels and may also be effective at scavenging free radicals. The beneficial effects of SA observed in the present study are in support of the findings of Rashedinia et al., 2020, Rashedinia et al., 2021, Manjunatha et al., 2020, and Sabahi et al., 2020, in other diabetic rat models [33,55,56,147]. Histopathological observations for kidney, sciatic nerve, liver, and heart and pathological injury scores are shown in Figure 6, Figure 7, Figure 8, Figure 9 and Figure 10.

## 4. Conclusions

SA appears to have antioxidative, anti-inflammatory, antihyperglycaemic, and antihyperlipidaemic properties; it was found to protect against the neuronal, cardiac, hepatic, and renal damage caused by chronic hyperglycaemia in Wistar rats. As a result, SA may be used to prevent T2DM-associated complications and/or organ damage. It may be combined with currently available antihyperglycaemic medications to reduce their dosage, prevent undesirable side effects, and delay the onset of tolerance. However, additional research on the mechanisms of action via gene expression studies and target-specific studies is required.

## Figures and Tables

**Table 1:** Effect of 10 weeks of repeated dose treatment of SA on body weight, food intake, water intake, and urine output of rats with nSTZ-induced T2DM at the end of the 8th and 18th weeks.

Group	Body Weight (gm)			Food Intake (gm)				Water Intake (mL)				Urine Output (mL)			
	8th Week	18th Week	% Increase in BW	8th Week	FI per 100 g b.w	18th Week	FI per 100 g b.w	8th Week	WI per 100 g b.w	18th Week	WI per 100 g b.w	8th Week	UOP per 100 g b.w	18th Week	UOP per 100 g b.w
NC	198.43 ± 12.15	271.17 ± 2.28	36.86 ± 6.25	9.66 ± 1.10	4.86 ± 0.30	14.83 ± 1.05	5.47 ± 0.29	19.57 ± 2.48	9.88 ± 1.29	29.98 ± 3.29	11.06 ± 1.19	2.14 ± 0.53	1.09 ± 0.31	3.12 ± 1.00	1.15 ± 0.37
NC + SA	187.96 ± 9.65	267.97 ± 10.0	42.86 ± 8.84	9.77 ± 0.71	5.21 ± 0.54	14.96 ± 1.40	5.59 ± 0.64	19.91 ± 1.72	10.66 ± 1.45	30.76 ± 3.20	11.50 ± 1.40	2.35 ± 0.54	1.25 ± 0.26	3.53 ± 0.98	1.31 ± 0.35
DC	186.56 ± 15.66	212.7 ± 17.96 ^C^	13.07 ± 3.64 ^C^	12.98 ± 1.09 ^C^	6.13 ± 0.66 ^B^	18.76 ± 1.08 ^C^	8.88 ± 1.09 ^C^	24.97 ± 4.59 ^B^	13.31 ± 1.47 ^C^	36.56 ± 4.33 ^B^	17.18 ± 1.34 ^C^	4.85 ± 1.145 ^C^	2.57 ± 0.44 ^C^	7.34 ± 1.34 ^C^	3.43 ± 0.43 ^C^
DC + SA25	200.84 ± 8.94	253.51 ± 10.96 ^d^	26.43 ± 7.65 ^a^	14.97 ± 0.89	5.90 ± 0.27 ^B^	17.86 ± 1.29	7.05 ± 0.57 ^Ad^	24.79 ± 1.77 ^A^	12.36 ± 1.07 ^A^	35.47 ± 2.91 ^A^	14.04 ± 1.64 ^Bb^	4.96 ± 0.67 ^C^	2.46 ± 0.22 ^C^	6.26 ± 0.81	2.47 ± 0.35 ^Cd^
DC + SA50	195.39 ± 14.40	252.52 ± 12.4 ^d^	29.46 ± 4.98 ^b^	15.04 ± 0.85	5.96 ± 0.27 ^B^	16.95 ± 1.60	6.70 ± 0.52 ^Ad^	24.02 ± 1.47 ^A^	12.33 ± 1.03 ^A^	32.92 ± 2.37	13.03 ± 0.69 ^d^	4.78 ± 0.96 ^C^	2.47 ± 0.59 ^C^	5.04 ± 0.78	1.99 ± 0.32 ^Bd^
DC + MET	189.77 ± 12.05	252.22 ± 9.41 ^d^	33.38 ± 9.98 ^d^	11.04 ± 1.27	5.82 ± 0.66	16.63 ± 1.57	6.59 ± 0.51 ^Ad^	23.41 ± 1.89	12.35 ± 0.82 ^A^	32.60 ± 1.78	12.92 ± 0.42 ^d^	4.66 ± 0.69 ^C^	2.45 ± 0.29 ^C^	4.80 ± 0.90	1.91 ± 0.39 ^Bd^

Data are expressed as mean ± SD, where *n* = 6 rats per group; A (*p* < 0.05), B (*p* < 0.01), and C (*p* < 0.001) indicate a significant difference from the normal control; a (*p* < 0.05), b (*p* < 0.01), and d (*p* < 0.001) indicate a significant difference from the diabetic control; and 1 (*p* < 0.05), 2 (*p* < 0.01), and 3 (*p* < 0.001) indicate a significant difference from the metformin-treated group. BW: body weight; FI: food intake; WI: water intake; UOP: urine output; NC: nondiabetic control; NC + SA: syringic acid (50 mg/kg, *p*.o. for 10 weeks) administered to nondiabetic control group; DC: n-STZ diabetic control; DC + SA25: syringic acid (25 mg/kg, p.o. for 10 weeks) administered to diabetic animals; DC + SA50: syringic acid (50 mg/kg, p.o. for 10 weeks) administered to diabetic animals; and DC + MET: metformin (200 mg/kg/day p.o. for 10 weeks) administered to diabetic animals.

**Table 2:** Effect of 10 weeks of repeated dose treatment of SA on blood biochemical estimations in rats with nSTZ-induced T2DM at the end of the 8th, 13th, and 18th weeks.

Parameters	No. of Weeks	NC	NC + SA	DC	DC + SA25	DC + SA50	DC + MET
FBG (mg/dL)	8	95.39 ± 3.56	92.75 ± 4.64	156.38 ± 4.40 ^C^	157.46 ± 5.38 ^C^	155.97 ± 7.76 ^C^	156.69 ± 6.67 ^C^
	13	91.54 ± 4.58	87.99 ± 5.29	204.32 ± 7.65 ^C^	183.59 ± 11.66 ^Cb2^	175.52 ± 6.53 ^Cd^	163.79 ± 8.41 ^Cd^
	18	91.02 ± 6.42	94.33 ± 4.85	247.86 ± 15.0 ^C^	218.96 ± 12.21 ^Cb3^	185.91 ± 10.34 ^Cd1^	165.00 ± 11.49 ^Cd^
BUN (mg/dL)	8	18.87 ± 3.54	19.83 ± 4.64	28.27 ± 4.41 ^B^	26.77 ± 5.58 ^A^	23.37 ± 4.29	25.68 ± 4.46
	13	20.04 ± 3.57	21.87 ± 3.91	38.75 ± 6.11 ^C^	37.13 ± 6.05 ^C^	28.58 ± 3.71 ^a^	29.13 ± 4.57 ^a^
	18	19.03 ± 2.26	22.34 ± 4.70	50.04 ± 5.48 ^C^	40.67 ± 6.95 ^Ca3^	34.69 ± 5.45 ^Cd^	31.07 ± 5.38 ^Bd^
SCr (mg/dL)	8	0.45 ± 0.050	0.43 ± 0.049	0.49 ± 0.089	0.45 ± 0.090	0.48 ± 0.047	0.47 ± 0.079
	13	0.49 ± 0.052	0.47 ± 0.075	0.81 ± 0.074 ^C^	0.54 ± 0.067 ^d^	0.53 ± 0.061 ^d^	0.53 ± 0.065 ^d^
	18	0.47 ± 0.064	0.45 ± 0.40	0.97 ±0.072 ^C^	0.60 ± 0.074 ^Ad^	0.57 ± 0.053 ^d^	0.55 ±0.11 ^d^
TC (mg/dL)	8	121.69 ± 6.27	113.52 ± 9.26	142.19 ± 5.39 ^C^	138.49 ± 6.43 ^C^	137.76 ± 7.74 ^B^	133.32 ± 7.12 ^A^
	13	115.92 ± 7.66	118.43 ± 10.95	194.01 ± 7.93 ^C^	172.59 ± 5.82 ^Cd3^	152.50 ± 5.45 ^Cd^	139.46 ± 6.95 ^Cd^
	18	117.41 ± 10.10	122.55 ± 8.45	259.83 ± 16.59 ^C^	213.14 ± 18.33 ^Cd3^	176.71 ± 15.18 ^Cd1^	148.67 ± 13.82 ^Ad^
TG (mg/dL)	8	79.84 ± 5.58	83.12 ± 4.86	92.2 ± 7.13 ^A^	95.03 ± 6.30 ^B^	88.28 ± 6.96	87.60 ± 7.79
	13	76.8 ± 6.64	82.01 ± 8.44	119.39 ± 10.31 ^C^	108.16 ± 5.49 ^C2^	93.75 ± 5.40 ^Bd^	91.79 ± 8.69 ^Ad^
	18	78.22 ± 5.41	81.54 ± 6.31	154.03 ± 6.07 ^C^	129.63 ± 11.35 ^Cb3^	98.46 ± 10.09 ^Ad^	92.47 ± 7.84 ^d^
AST (U/L)	8	97.63 ± 8.99	103.18 ± 5.21	114.66 ± 7.72 ^A^	117.45 ± 7.87 ^B^	116.47 ± 9.57 ^B^	113.13 ± 6.59 ^A^
	13	103.64 ± 4.55	105.93 ± 7.01	147 ± 12.85 ^C^	141 ± 8.29 ^C3^	124.78 ± 11.60 ^Bb^	117.32 ± 5.87 ^d^
	18	104.36 ± 7.54	107.79 ± 11.38	185.53 ± 13.39 ^C^	160.63 ± 17.56 ^Ca3^	138.49 ± 10.13 ^Bd^	121.17 ± 12.85 ^d^
ALT (U/L)	8	39.81 ± 5.20	37.93 ± 4.18	53.12 ± 5.96 ^B^	52.36 ± 3.89 ^B^	54.56 ± 5.16 ^B^	53.4 ± 6.25 ^B^
	13	40.88 ± 4.71	39.66 ± 4.24	59.71 ± 3.72 ^C^	55.85 ± 5.66 ^C^	52.63 ± 6.25 ^A^	49.51 ± 5.93 ^a^
	18	41.23 ± 3.16	42.57 ± 4.47	70.93 ± 5.11 ^C^	61.76 ± 6.35 ^Ca3^	51.28 ± 5.28 ^Ad^	45.66 ± 4.36 ^d^

Data are expressed as mean ± SD, where *n* = 6 rats per group; A (*p* < 0.05), B (*p* < 0.01), and C (*p* < 0.001) indicate a significant difference from the normal control; a (*p* < 0.05), b (*p* < 0.01), and d (*p* < 0.001) indicate a significant difference from the diabetic control; and 1 (*p* < 0.05), 2 (*p* < 0.01), and 3 (*p* < 0.001) indicate a significant difference from the metformin-treated group. FBG: fasting blood glucose; SCr: serum creatinine; BUN: blood urea nitrogen; TG: triglyceride; TC: total cholesterol; ALT: alanine transaminase; AST: aspartate transaminase; NC: nondiabetic control; NC + SA: syringic acid (50 mg/kg, p.o. for 10 weeks) administered to nondiabetic control group; DC: n-STZ diabetic control; DC + SA25: syringic acid (25 mg/kg, p.o. for 10 weeks) administered to diabetic animals; DC + SA50: syringic acid (50 mg/kg, p.o. for 10 weeks) administered to diabetic animals; and DC + MET: metformin (200 mg/kg/day p.o. for 10 weeks) administered to diabetic animals.

**Table 3:** Effect of 10 weeks of repeated dose treatment of SA on urinary biochemistry in rats with nSTZ-induced T2DM at the end of the 18th week.

Groups	UCr (mg/dL)	CrCl (ml/min)	UALB (mg/L)	Na (mEq/L)	K (mEq/L)
NC	38.26 ± 2.09	270.46 ± 57.03	20.37 ± 8.37	104.70 ± 16.75	22.50 ± 3.54
NC + SA	36.12 ± 5.16	285.70 ± 77.95	18.51 ± 5.73	105.28 ± 7.59	20.31 ± 4.32
DC	16.35 ± 2.30 ^C^	124.30 ± 26.30 ^C^	127.22 ± 33.13 ^C^	156.61 ± 13.82 ^C^	8.26 ± 2.88 ^C^
DC + SA 25	23.66 ± 2.88 ^Ca^	217.01 ± 30.17 ^a^	86.11 ± 17.84 ^Cb2^	126.67 ± 12.33 ^b^	14.32 ± 2.62 ^Ba^
DC + SA50	29.50 ± 4.85 ^Bd^	242.83 ± 40.65 ^b^	56.85 ± 14.54 ^Bd^	114.90 ± 10.96 ^d^	17.13 ± 2.48 ^Ad^
DC + MET	30.80 ± 3.15 ^Ad^	254.05 ± 27.75 ^d^	45.18 ± 8.65 ^d^	108.47 ± 11.15 ^d^	18.02 ± 2.26 ^d^

Data are expressed as mean ± SD, where *n* = 6 rats per group; A (*p* < 0.05), B (*p* < 0.01), and C (*p* < 0.001) indicate a significant difference from the normal control; a (*p* < 0.05), b (*p* < 0.01), and d (*p* < 0.001) indicate a significant difference from the diabetic control; and 1 (*p* < 0.05), 2 (*p* < 0.01), and 3 (*p* < 0.001) indicate a significant difference from the metformin-treated group.

**Table 4:** Effect of 10 weeks of repeated dose treatment of SA on blood biochemical estimations in rats with nSTZ-induced T2DM at the end of the 18th week.

Parameters	NC	NC + SA	DC	DC + SA25	DC + SA50	DC + MET
HbA1C (%)	4.32 ± 0.70	4.52 ± 0.81	7.881 ± 0.60 ^C^	6.38 ± 1.02 ^Ba^	5.89 ± 0.64 ^Ab^	5.17 ± 0.78 ^d^
TG (mg/dL)	85.32 ± 6.43	89.10 ± 7.00	259.83 ± 16.59 ^C^	213.14 ± 18.33 ^Cd3^	176.71 ± 15.18 ^Cd1^	148.67 ± 13.82 ^Ad^
TC (mg/dL)	117.41 ± 10.10	122.55 ± 8.45	154.03 ± 6.81 ^C^	129.68 ± 11.80 ^Cb3^	98.46 ± 9.58 ^Ad^	92.47 ± 7.40 ^d^
HDL (mg/dL)	50.33± 6.03	54.16 ± 4.52	32.75± 3.46 ^C^	42.50± 6.16 ^a^	44.95 ± 4.90 ^b^	46.16 ± 4.49 ^b^
LDL (mg/dL)	49.99± 12.24	50.57± 10.30	196.26 ± 16.27 ^C^	144.72± 8.80 ^Cd3^	112.09 ± 16.53 ^Cd^	83.96± 16.60 ^Ad^
AI	1.36 ± 0.35	1.27 ± 0.27	6.98 ± 0.77	4.10 ± 0.85 ^Ab^	2.97 ± 0.58 ^d^	2.26 ± 0.59 ^d^
TP (g/dL)	8.56 ± 0.71	9.20 ± 0.64	5.55 ± 0.42 ^C^	6.85 ± 0.43 ^Cb1^	7.38 ± 0.43 ^Ad^	7.89 ± 0.52 ^d^
SCr (mg/dL)	0.47 ± 0.06	0.45 ± 0.07	0.97 ± 0.19 ^C^	0.68 ± 0.11 ^Bd^	0.61 ± 0.08 ^d^	0.57± 0.07 ^d^
BUN (mg/dL)	19.03 ± 2.19	22.34 ± 3.46	50.04 ± 5.64 ^C^	37.67 ± 4.47 ^Cb1^	32.67 ± 5.64 ^Cd^	27.56 ± 4.68 ^Ad^
ALB (g/dL)	3.72 ± 0.41	3.84 ± 0.61	2.66 ± 0.45 ^C^	3.17 ± 0.12 ^Aa1^	3.51 ± 0.31 ^d^	3.67 ± 0.12 ^d^
CRP (mg/l)	0.60 ± 0.65	0.8 ± 0.61	3.60 ± 1.31 ^C^	2.20 ± 0.48 ^Aa^	1.80 ± 0.65 ^d^	1.6 ± 0.61 ^d^
LDH (U/l)	339.53 ± 37.69	345.89 ± 45.70	698.02 ± 56.87 ^C^	575.84 ± 26.97 ^Cd3^	439.68 ± 43.95 ^Bd^	415.34 ± 36.86 ^d^
CK-MB (U/l)	294.41 ± 25.11	301.58 ± 24.73	507.21 ± 62.19 ^C^	438.56 ± 23.76 ^Ca3^	346.76 ± 25.72 ^d^	325.56 ± 37.82 ^d^
AST (U/L)	104.36 ± 7.54	107.79 ± 11.38	185.53 ± 13.39 ^C^	160.63 ± 17.22 ^Ca3^	138.49 ± 10.13 ^Cd^	121.17 ± 12.85 ^d^
ALT (U/L)	41.23 ± 3.16	42.57 ± 4.47	70.93 ± 5.11 ^C^	61.76 ± 6.35 ^Ca3^	51.28 ± 5.28 ^Ad^	45.66± 4.36 ^d^
BR (mg/dL)	0.25 ± 0.04	0.24 ± 0.07	0.46 ± 0.04 ^C^	0.37 ± 0.04 ^Ca2^	0.32 ± 0.06 ^d^	0.26 ± 0.043 ^d^

Data are expressed as mean ± SD, where *n* = 6 rats per group; A (*p* < 0.05), B (*p* < 0.01), and C (*p* < 0.001) indicate a significant difference from the normal control; a (*p* < 0.05), b (*p* < 0.01), and d (*p* < 0.001) indicate a significant difference from the diabetic control; and 1 (*p* < 0.05), 2 (*p* < 0.01), and 3 (*p* < 0.001) indicate a significant difference from the metformin-treated group. HbA1C: glycated haemoglobin; TG: triglyceride; TC: total cholesterol; HDL: high-density lipoprotein; LDL: low-density lipoprotein; AI: atherogenic index; TP: total protein; SCr: serum creatinine; BUN: blood urea nitrogen; ALB: albumin; CRP: C-reactive protein; LDH: lactate dehydrogenase; CK-MB: Creatinine Kinase-MB; ALT: alanine transaminase; AST: aspartate transaminase; BR: bilirubin; NC: nondiabetic control; NC + SA: syringic acid (50 mg/kg, p.o. for 10 weeks) administered to nondiabetic control group; DC: n-STZ diabetic control; DC + SA25: syringic acid (25 mg/kg, p.o. for 10 weeks) administered to diabetic animals; DC + SA50: syringic acid (50 mg/kg, p.o. for 10 weeks) administered to diabetic animals; and DC + MET: metformin (200 mg/kg/day p.o. for 10 weeks) administered to diabetic animals.

**Table 5:** Effect of 10 week repeated dose treatment of SA on gastric emptying and small intestinal transit in rats with nSTZ-induced T2DM at the end of 18th week.

Group	% GE	% SIT
NC	53.54 + 8.89	63.12 + 5.37
NC+SA	49.10 + 4.10	64.78 + 4.38
DC	25.89 + 8.98 ^C^	36.89 + 2.66 ^C^
DC + SA25	41.47 + 7.16 ^a^	48.04 + 5.17 ^Cb1^
DC + SA50	45.38 + 4.22 ^d^	53.77 + 4.87 ^Ad^
DC + MET	48.38 + 6.10 ^d^	57.78 + 4.77 ^d^

Data are expressed as mean ± SD, where *n* = 6 rats per group; A (*p* < 0.05), B (*p* < 0.01), and C (*p* < 0.001) indicate a significant difference from the normal control; a (*p* < 0.05), b (*p* < 0.01), and d (*p* < 0.001) indicate a significant difference from the diabetic control; and 1 (*p* < 0.05), 2 (*p* < 0.01), and 3 (*p* < 0.001) indicate a significant difference from the metformin-treated group. % GE: percent gastric emptying; %SIT: percent small intestinal transit; NC: nondiabetic control; NC + SA: syringic acid (50 mg/kg, p.o. for 10 weeks) administered to nondiabetic control group; DC: n-STZ diabetic control; DC + SA25: syringic acid (25 mg/kg, p.o. for 10 weeks) administered to diabetic animals; DC + SA50: syringic acid (50 mg/kg, p.o. for 10 weeks) administered to diabetic animals; and DC + MET: metformin (200 mg/kg/day p.o. for 10 weeks) administered to diabetic animals.

## Data Availability

Data are contained within the article.
